# Protective effects for HLA-B*40:01 and C*03:04 in NPM1-mutated AML: result of a large HLA association study

**DOI:** 10.3389/fimmu.2025.1571508

**Published:** 2025-06-10

**Authors:** Elke Rücker-Braun, Bose Falk, Henning Baldauf, Carolin Massalski, Gesine Schäfer, Heidi Altmann, Jürgen Sauter, Ute V. Solloch, Vinzenz Lange, Katharina Egger-Heidrich, Desiree Kunadt, Friedrich Stölzel, Christoph Röllig, Jan M. Middeke, Malte von Bonin, Christian Thiede, Alexander H. Schmidt, Martin Bornhäuser, Johannes Schetelig, Falk Heidenreich

**Affiliations:** ^1^ Department of Internal Medicine I, University Hospital TU Dresden, Dresden, Germany; ^2^ Clinical Trials Unit, DKMS Group, Dresden, Germany; ^3^ DKMS Life Science Lab, Dresden, Germany; ^4^ DKMS Group, Tübingen, Germany; ^5^ Division for Stem Cell Transplantation and Cellular Immunotherapy, Department of Medicine II, University Hospital Schleswig-Holstein, Kiel, Germany

**Keywords:** AML, NPM1, *DNMT3A*, mutation, HLA association study

## Abstract

**Introduction:**

Mutations in the nucleophosmin 1 gene (NPM1) are common and recurrent molecular abnormalities in acute myeloid leukemia (AML). NPM1 mutations are considered to be positive prognostic factors. The beneficial effect may be due to immune responses mediated by cytotoxic T cells targeting HLA-presented peptides derived from mutated NPM1 and thereby suppressing mutated NPM1-positive hematopoiesis. While the immunogenicity of these NPM1 peptides has not been demonstrated conclusively, certain HLA-types have been linked to a lower risk of NPM1-mutated AML.

**Method:**

In a comprehensive HLA association study at two-field resolution, we compared the proportions of HLA class I alleles between NPM1-mutated (n = 477) and/or DNMT3A-mutated (n = 216) patients with AML and a control group of healthy individuals (n = 51,890).

**Result:**

We found HLA-B*40:01 and HLA-C*03:04 to be significantly underrepresented in NPM1-mutated AML compared to the control group (4.0% vs. 10.2%, p < 0.001, and 8.2% vs, 15.9%, p < 0.001, respectively). This might suggest that neoepitopes presented by these HLA alleles trigger T-cell responses. Online epitope prediction tools predict that mutated NPM1-derived peptides bind strongly to B*40:01 and C*03:04.

**Discussion:**

Based on these findings, further studies should confirm the presence and functionality of neoepitope-specific T cells and characterize specific T-cell receptors (TCR). Sequence information might eventually be exploited in immunotherapeutic approaches to treat AML patients with TCR-engineered T cells or bispecific TCR T/NK cell engagers.

## 1 Introduction

The immune system is believed to have the potential to eradicate leukemia cells during development of acute myeloid leukemia (AML). AML has a low mutational burden in comparison to solid tumors, but some high-incidence driver mutations result in immunogenic peptides making AML vulnerable to assault by the adaptive immune systems (1).

*Nucleophosmin 1 (NPM1)* is the most frequently mutated (2) gene in AML, and up to 60% of adult patients with normal karyotype harbor a mutation in the *NPM1* gene (3). NPM1 mutations in AML define a distinct leukemia entity [WHO classification 2022 and ICC 2022: AML-defining recurrent genetic abnormalities], can act as founder mutations that are stable over the course of disease (3–6), and are not expressed in normal tissue (7). The presence of mutated NPM1 (NPM1mut) is considered to be a positive prognostic factor (8). The favorable clinical outcome of AML patients with NPM1mut might be associated with immune responses mediated by NPM1mut-specific CD8+ T cells (9).

Mutated *NPM1* is a driver gene that is essential for malignant transformation early in the development of leukemia. The *NPM1* gene, mapping to chromosome 5q35.1 in humans, contains 13 exons (10). The wild-type NPM1 is a 294-amino acid (aa) ubiquitous and multifunctional nucleocytoplasmic shuttling phosphoprotein. NPM1 plays a crucial role in cell proliferation, survival, and nucleic acid metabolism. It is located primarily in nucleoli, but shuttles continuously between nucleolus and cytoplasm (11, 12). Some mutations in *NPM1* arise from the insertion or duplication of four to nine nucleotides. These mutations typically involve exon 12 of the *NPM1* gene resulting in the impairment of the nucleolar localization signal and the addition of a nuclear export signal (13). Consequently, the mutated NPM1 protein is predominantly located in the cytoplasm. Although 55 frame shift mutations have been reported, the three most common ones are mutations A, B, and D accounting for 75%–80%, approximately 10%, and approximately 5% of all NPM1-mutated AML cases, respectively (3, 7, 14). Notably, the *NPM1* mutations A and D result in the same aa sequence change.

NPM1-mutated AML is usually accompanied by concomitant mutations, including *DNA-methyltransferase 3A (DNMT3A)*. DNMT3A mutations occur in 15%–25% of adult AML patients and up to 36% of adults with intermediate-risk normal cytogenetic AML harbor a mutation in DNMT3A (12) and are early hits in hematopoietic stem cells (15, 16). One mutation hotspot impacts the arginine at the aa position 882 (R882). Different R882 mutations are documented, with the substitution by histidine R882H being the most frequent (~60%), followed by the substitution by cysteine R882C (24%–36%) (17).

DNMT3A is a 130-kDa protein encoded by 23 exons located in chromosomal band 2p23. *DNMT3A* encodes an enzyme that is responsible for the transfer of a methyl group to cytosine in CpG dinucleotide residues, which are concentrated in the promotor regions of genes. Increased methylation of these CpG islands is often associated with reduced expression of the downstream gene. The affected genes involve tumor-suppressor genes, and aberrant DNA methylation has been hypothesized to contribute to the pathogenesis of cancer. It has been shown that R882 mutations result in decreased catalytic activity and that the mutations contribute to AML pathogenesis as a key initiating event (18). It is worth noting that DNMT3A-mutated hematopoietic cells should not be considered as malignant cells *per se* but as pre-leukemic cells, which could cause and host the final AML (17, 19, 20).

Sparse data on the HLA-restriction pattern of NPM1mut and DNMT3A R882H-mutated AML are available and indicate a role of the immune system during AML leukemogenesis and throughout the course of the disease (21, 22). Kuzelova et al. (2018) could identify significant differences in HLA class I profiles when comparing AML patients with NPM1 mutation and healthy individuals who indirectly support the hypothesis that T-cell-mediated immune responses contribute to disease control in patients with NPM1 mutations (23). Therefore, we conducted a large HLA class I allele association study for NPM1mut and DNMT3A R882H-mutated AML at two-field resolution to confirm the concept that immune reactions against mutated NPM1 and/or DNMT3A can prevent AML development. Herewith, we provide further evidence that there is a natural T-cell-mediated graft-versus-leukemia (GvL) effect. Such immunological insights can be exploited for T-cell receptor-based immunotherapy to eradicate leukemic and pre-leukemic clones.

## 2 Materials and methods

### 2.1 Samples from patients and donors

We conducted a large association study on the impact of HLA class I alleles on patients with AML who donated for Collaborative Biobank (24) (www.cobi-biobank.de) or Study Alliance Leukemia (SAL) Biobank. Patients were selected by the following eligibility criteria: patient has diagnosis of AML or secondary AML following MDS, does not have a diagnosis of treatment-related myeloid neoplasia and was enrolled by a German registry site. All 1,689 patients, whose samples were analyzed in this study, had provided written informed consent. Among the 477 AML patients with mutations A/D of NPM1, 90 patients were also analyzed by Kuzelova et al. (2018). As a reference group, 51,890 recently HLA-typed potential unrelated stem cell donors by DKMS Germany had been used. The study was approved by the Ethical Committee of the TU Dresden (EK 249072018).

### 2.2 Detection of *NPM1* exon 12 mutations, *DNMT3A* exon 23 mutations, and HLA genotyping

Sequencing for *NPM1* mutations was performed as described previously (25). Briefly, polymerase chain reaction (PCR) amplification of *NPM1* exon 12 was carried out using the published primer molecules NPM1-F and NPM1-R. PCR products were sequenced with primer NPM1-R2 using ABI Ready Reaction Dye Terminator Cycle Sequencing Kit (Applied Biosystems).

*DNMT3A* mutations were analyzed by PCR of genomic DNA and direct sequencing with primers spanning exon 23. PCR reactions were performed on a GeneAmp PCR System 9700 (Applied Biosystems, Foster City, CA, USA) as described previously (26).

At the time of stem cell donor registration, volunteers provided buccal swabs for DNA extraction and genotyping. The genotyping of HLA classes I and II loci (HLA-A, -B, -C, -DRB1, -DPB1 and -DQB1) was performed by the DKMS Life Science Lab applying a high-resolution amplicon-based approach using Illumina devices (27). The DNA of patients and donors had been typed with the same workflow.

### 2.3 Statistical analysis

**Figure 1:**
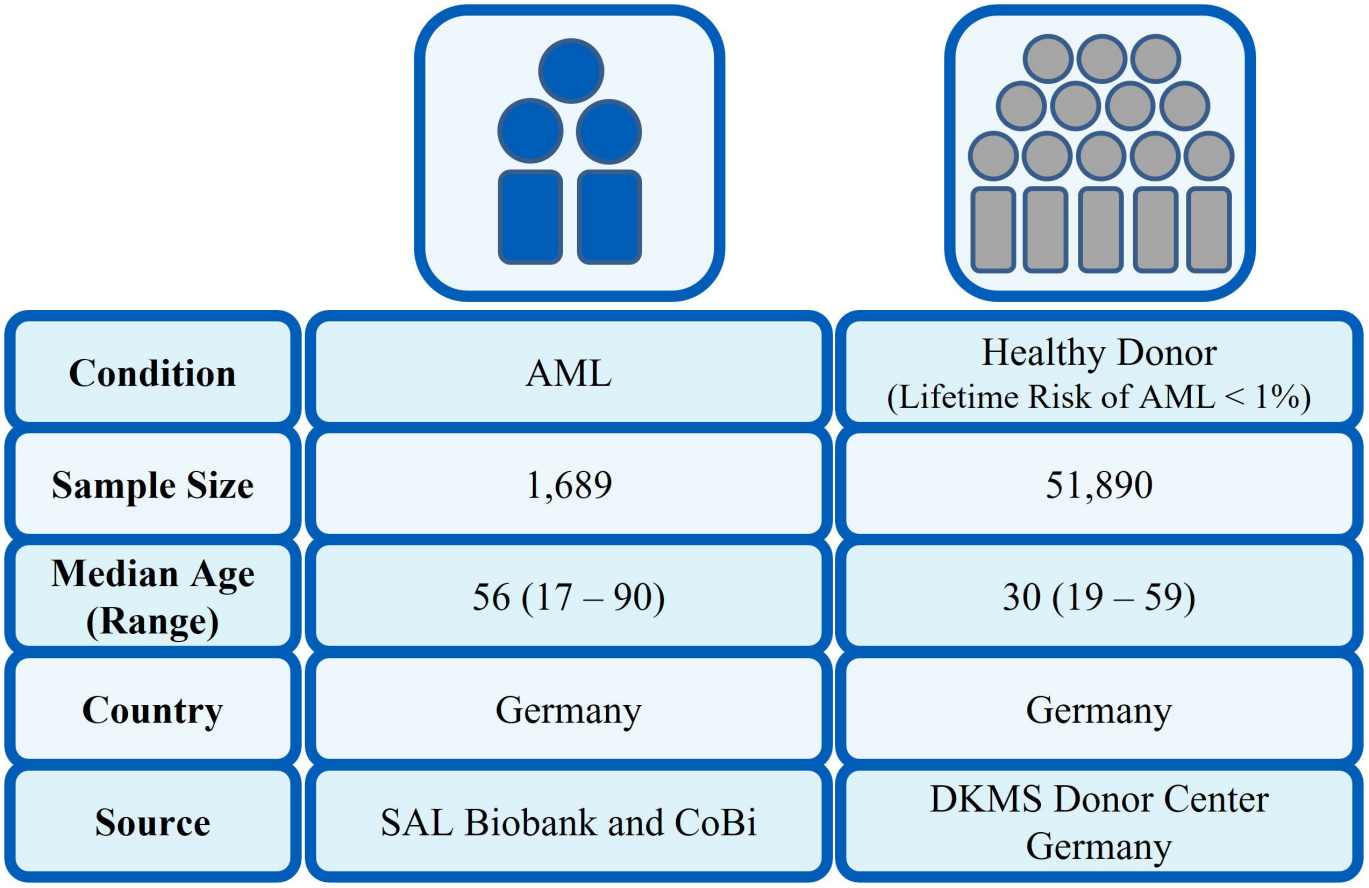
Characteristics of patient and control cohort.

The analysis set contained information on 1,689 patients and 51,890 healthy individuals (**Figure 1**). For each allele in the class I allelic groups, the proportion of patients expressing this allele was compared with the proportion in the healthy control group. This proportion was tested using a two-sided z-test—as an illustration of the method, considering HLA-A*24:02 where 95 out of 477 NPM1-mutated patients had this allele (19.9%), the healthy control group had 8,965 out of 51,890 (17.3%), giving a two-sided z-test p-value of 0.145. For the figures where we simultaneously compare the top 10 alleles for each HLA locus, the printed p-value has been adjusted for multiple testing by Bonferroni correction; otherwise, the un-adjusted p-value is reported. p-Values are only printed on the graph if they are <0.05.

### 2.4 Immunopeptide prediction analysis *in silico*

We used different *in silico* epitope prediction software tools (NetMHCpan version 4.1b, SYFPEITHI, RankPep, and IEDB) to identify candidate immune-epitope peptides derived from either mutated NPM1 or mutated DNMT3A sequences to bind common HLA class I molecules. NetMHCpan-4.1b were made using the IEDB analysis resource Consensus tool (28), which combines predictions from ANN aka NetMHC (4.0) (29), SMM (30), and Comblib (31). Predictions were selected on four peptide lengths, octamer (8-mer), nonamer (9-mer), decamer (10-mer), and undecamer (11-mer). NetMHCpan-4.1b defined peptides with a percentile rank below 2% as weak binders and below 0.5% as strong binders (32). For SYFPEITHI, peptides scoring above 20 are highly likely to bind. High binding affinities of peptides to MHC I are assumed when scores of RankPep are higher than the binding threshold (values in brackets). For IEDB, a low percentile rank represents a good binder.

## 3 Results

In our population-based case-control study, we compared HLA class I frequencies at two-field resolution in a large cohort of AML patients (n = 1,689) with a control group of 51,890 healthy individuals. The basic parameters of patients with AML are given in **Table 1**. For the healthy control group, the median age was 30 years with a range of 19 to 59 years. In total, 65.9% were female and 34.1% were male.

**Table 1:** AML patient cohort characteristics.

Demographic data	
Median age (range)*	56 (17–90)
Sex*	
Female	49.5% (487)
Male	50.5% (503)
Country	German

Biobank	
CoBi	698
SAL	990

Disease classification*	
AML status	
*De novo* AML	87.6% (867)
sAML	11.6% (115)
tAML	0.8% (8)
ELN risk category	
Favorable	35.2% (349)
Intermediate	59.6% (589)
Adverse	5.0% (50)
n.a.	0.2% (2)
ECOG	
0	20.7% (205)
1	51.6% (511)
2	16.6% (164)
3	3.9% (39)
4	1.5% (15)
n.a.	5.7% (57)

*Data are only available for SAL biobank samples. The absolute numbers are given in brackets.

### 3.1 Underrepresentation of HLA-B*40:01 and HLA-C*03:04 in AML patients

**Figure 2:**
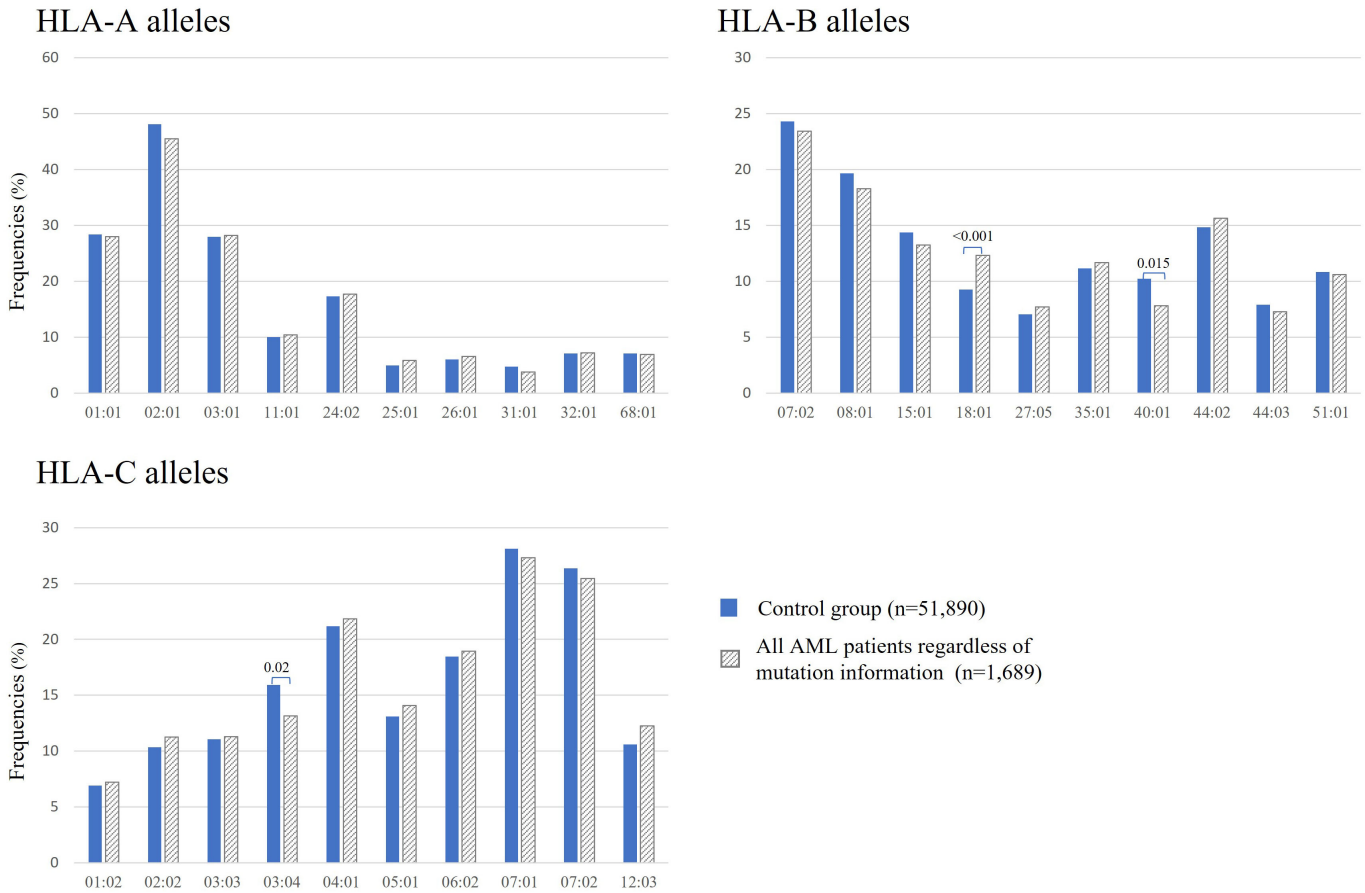
Frequencies of the 10 most frequent HLA-A, -B, and -C alleles in all patients with AML regardless of mutation status (gray bars) and in healthy donors (blue bars). Significant adjusted p-values are depicted above the columns.

**Figure 2** shows the observed frequencies of the 10 most frequent HLA-A, -B, and -C alleles, respectively, in patients with AML and in healthy individuals. In AML patients regardless of mutation status, statistically significant reductions of frequencies were observed for the two following HLA alleles: B*40:01 (n = 132 of 1.689, 7.8% vs, 10.2%, p = 0.015) and C*03:04 (n = 222 of 1.689, 13.1% vs. 15.9%, p = 0.024) when compared to the control group. Interestingly, the frequency for B*18:01 (n = 208 of 1.689, 12.3% vs. 9.2%, p < 0.001) is significantly increased in this AML cohort when compared to the control group (**Figure 2**).

Patients with AML were grouped according to their NPM1 or DNTMA3A mutation status. The NPM1 mutations A/D (NPM1mutA/D) were found in 477 patients, whereas 87 patients were positive for DNMT3A R882H. In 60 patients, both mutations have been identified (**Table 2**).

**Table 2:** Numbers of AML patients with NPM1 mutations and/or DNMT3A mutations.

Variant	NPM1mut A/D	NPM1 rare mut	No NPM1 mutation	NPM1 status not available	Total
DNMT3A R882H	60	5	22	0	**87**
DNMT3A R882C	24	4	9	0	**37**
DNMT3A rare mut	41	13	38	0	**92**
No DNMT3A mutation	61	26	147	0	**234**
DNMT3A status not available	291	57	374	517	**1,239**
Total	**477**	**105**	**590**	**517**	**1,689**

Bold values are total numbers.

### 3.2 Protective effect of HLA-B*40:01 and C*03:04 in AML patients with NPM1 mutations A/D

**Figure 3:**
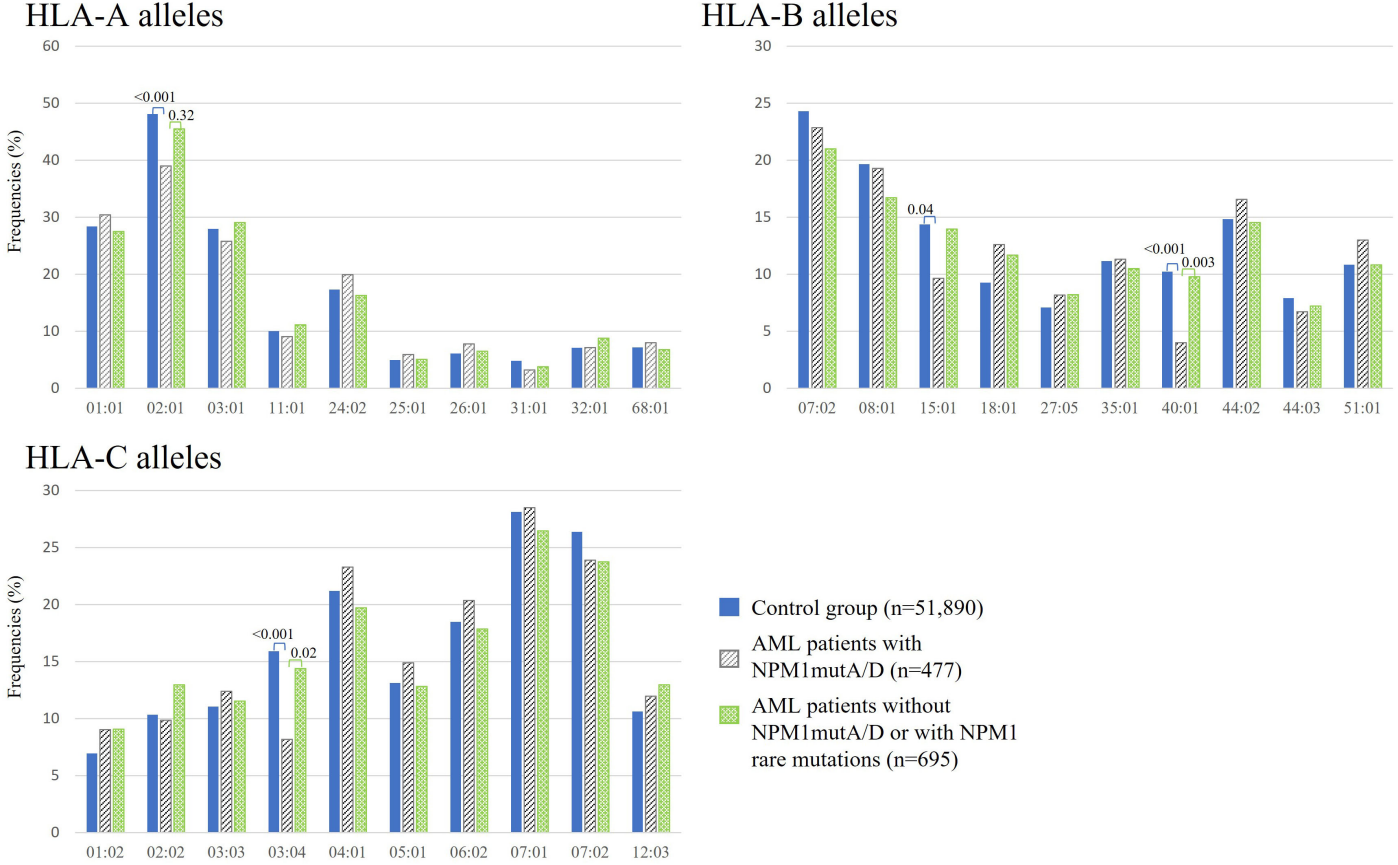
Frequencies of the 10 most frequent HLA-A, -B, and -C alleles in healthy donors (blue bars), AML patients with NPM1mutA/D (gray bars), and without NPM1mutA/D or with NPM1 rare mutations (green bars). p-Values were adjusted and are depicted above the columns.

By focusing on the NPM1 or DNMT3A mutation status in the AML patient cohort, we compared HLA class I profiles. **Figure 3** shows the observed HLA class I allele frequencies in AML patients with NPM1mutA/D (n = 477), in AML patients without NPM1 mutation (w/o NPM1mut) or with rare NPM1 mutations (NPM1mut^rare^) (n = 695) and in the control group of 51,890 healthy individuals. Compared with healthy donors, the incidence of the HLA alleles A*02:01 (n = 186, 39.0% vs. 48.1%, p < 0.001), B*15:01 (n = 46, 9.6% vs. 14.4%, p = 0.041), B*40:01 (n = 19, 4.0% vs. 10.2%, p < 0.001), and HLA-C*03:04 (n = 39, 8.2% vs. 15.9%, p < 0.001) were found to be significantly decreased in AML patients with NPM1mutA/D. Even significantly decreased frequencies for HLA alleles B*40:01 and C03:04 were observed for AML patients with NPM1mutA/D (n = 477) compared with AML patients w/o NPM1mut or with NPM1mut^rare^ (n = 695). We could not identify a significantly skewed HLA class I allele distribution in DNMT3A R882H-mutated AML (n = 87), but reduced frequencies for HLA-B*40:01 and C*03:04 were also observed (**Supplementary Figure S1**). The same pattern was achieved when comparing AML patients with at least one of the two DNMT3A mutations: R882H and R882C (n = 124), with the healthy control group (**Supplementary Figure S2**).

### 3.3 What is the driver of immune surveillance?

**Figure 4:**
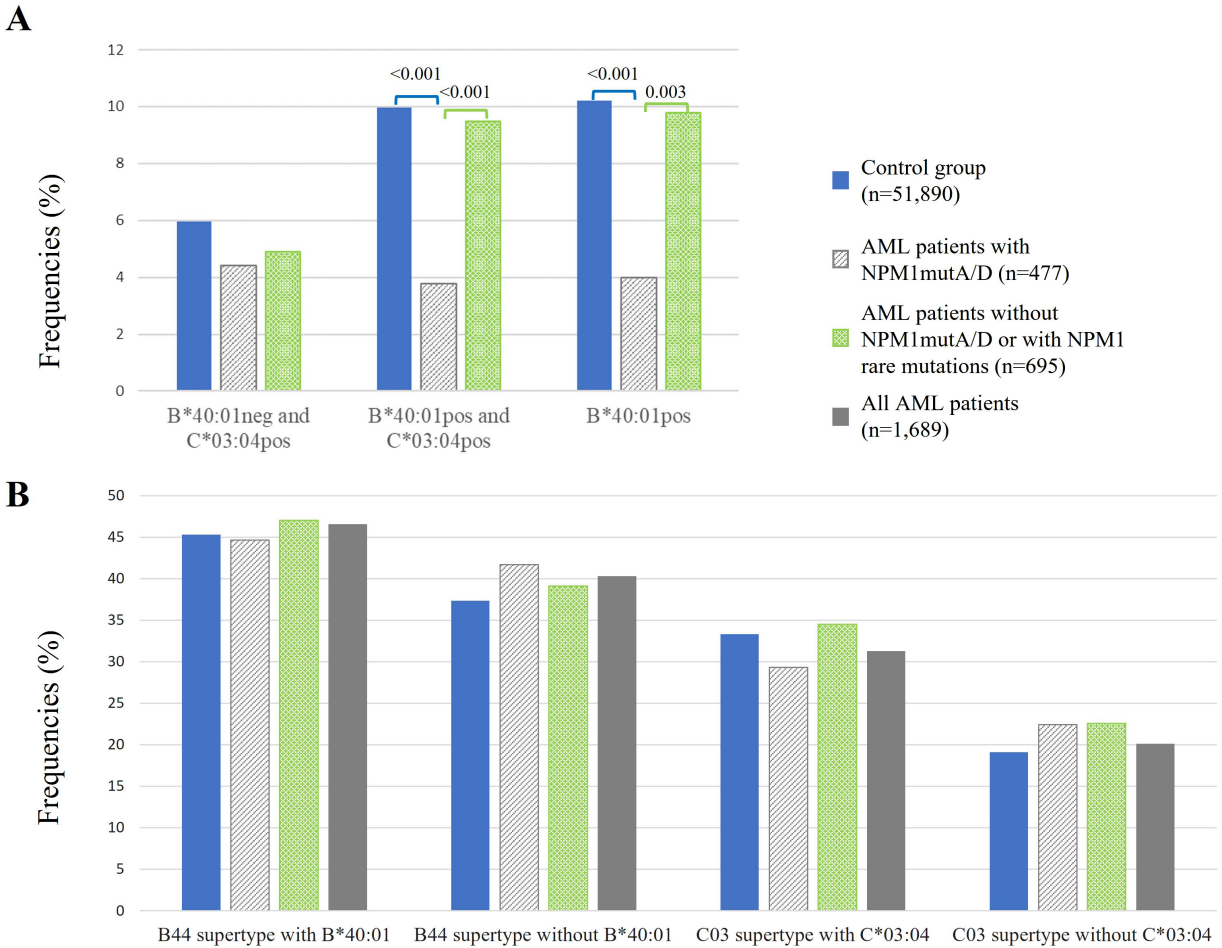
Frequencies of healthy control (blue bars) in comparison to AML patients (gray, green, and dark gray bars) among B*40:01-negative–C*03:04-positive, B*40:01-positive–C*03:04-positive, and B*40:01-positive individuals **(A)** and of HLA-B and HLA-C supertypes **(B)**. neg, negative; pos, positive. p-Values were adjusted and are depicted above the columns.

Relative linkage disequilibrium was shown for HLA-B*40:01 and HLA-C*03:04 based on a high-resolution HLA genotyping study of 8,862 German stem cell donors (33). Among this group, the most common linkage was HLA-B*40:01 to C*03:04, while HLA-C*03:04 links less frequently to B*40:01 (33). To investigate which HLA allele, HLA-B*40:01 or HLA-C*03:04, has the main influence on the protective effect, we compared frequencies of patients with AML and of the control group among B*40:01-negative–C*03:04-positive (B*40:01neg–C*03:04pos) (n = 21 out of 477), B*40:01-positive–C*03:04-positive (B*40:01pos–C*03:04pos) (n = 18 out of 477) in comparison with HLA-B*40:01-positive (B*40:01pos) (n = 19 out of 477) individuals as shown in **Figure 4A**. The HLA-B*40:01-positive–C*03:04-negative subgroup (n = 1 out of 477) was too small to analyze.

We could not find significant differences when comparing B*40:01neg–C*03:04posAML patients with NPM1mutA/D (n = 21 of 477) to the control group, or to AML patients w/o NPM1mutA/D, or with NPM1mut^rare^. However, the frequency of B*40:01pos–C*03:04pos among AML patients with NPM1mutA/D (n = 18 out of 477) was significantly reduced compared to healthy controls (3.8% vs. 10.0%, p < 0.001) and was also significantly reduced when compared to AML patients w/o NPM1mutA/D or with NPM1mut^rare^ (3.8% vs. 9.5%, p<0.001). Similarly, the frequency of B*40:01pos AML patients with NPM1mutA/D (n = 19 out of 477) was significantly reduced when compared to healthy controls (4.0% vs. 10.2%, p < 0.001) or to AML patients w/o NPM1mutA/D or with NPM1mut^rare^ (4.0% vs. 9.8%, p = 0.003) (**Figure 4A**).

### 3.4 Cluster of HLA peptide-binding motifs (PBM) and their protective effect

HLA alleles can be clustered based on their shared peptide-binding specificities defined as supertypes or based on a revised classification as cluster of HLA peptide-binding motifs (PBM). This grouping allows a more streamlined analysis of HLA effects. The HLA-B44 supertype or PBM8 cluster represents the following HLA-B alleles with highly similar PBMs: 18:01, 18:03, 40:01, 40:02, 41:01, 41:03, 44:02, 44:03, 44:27, 44:28, 45:01, 49:01, and 50:01 (34, 35). The HLA-C03 supertype or PBM18 cluster comprises HLA-C alleles 01:02, 03:04, 03:03, and 17:01 with similarity of peptide binding motif (36). For additional comparison analyses, B*40:01 and C*03:04 were omitted from the HLA-B44 supertype (PBM8-cluster) and the HLA-C03 supertype (PBM18 cluster), respectively.

We explored if the abovementioned HLA supertypes provide a protective effect. For HLA-B44, we excluded HLA-B*40:01, which has a protective effect, and for HLA-C03, we excluded HLA-C*03:04, which has a protective effect as well. Here, we distinguish the following three patient cohorts for analysis: AML patients with NPM1mutA/D (n = 477), AML patients w/o NPM1mutA/D or with NPM1mut^rare^ (n = 695), and all AML patients (n=1,689). We could not detect significant effects of HLA supertypes in the three patient cohorts (**Figure 4B**).

### 3.5 Candidates for the most immunogenic peptides from NPM1-mutated and DNMT3A R882H-mutated proteins

The underrepresentation of specific HLA class I alleles in AML patients with NPM1mutA/D suggests that individuals with specific HLA types may have more effective anti-NPM1 immune responses and are more resistant to AML development.

Four different algorithms were used to identify peptides derived from mutated NPM1 or mutated DNMT3A to bind selected HLA class I molecules with high affinity. For NPM1, we evaluated the last 21 C-terminal aa of mutations A/D and, for DNMT3A, the 20 aa surrounding position 882H of the mutated DNMT3A protein. Peptides were scored for their predicted binding affinity to the significantly underrepresented HLA class I alleles (A*02:01, B*40:01, B*15:01, and C*03:04) and the one overrepresented HLA-B*18:01. Strong binding peptides were found for B*40:01 and C*03:04 (QEAIQDLCL, LAVEEVSL derived from NPM1mut and MSHLARQRL derived from DNMT3Amut) using NetMHCpan-4.1b, and applying the MHC-I binding prediction tool of IEDB, the abovementioned peptides obtained the lowest percentile rank. Promising candidate peptides (AIQDLCLAV and CLAVEEVSL derived from NPM1mut) could be identified for A*02:01 by all four prediction algorithms (in NetMHCpan-4.1b as weak binders and with intermediate percentile ranks from IEDB, but with the highest total scores from SYFPEITHI). No prediction could be obtained from SYFPEITHI and RankPep for C*03:04, B*40:01, and B*18:01 with the exception of QEAIQDLCL. The peptides with the best scores (if any) are shown in **Table 3**. In summary, binding was predicted for a subset of neoantigen-derived peptides, particularly AIQDLCLAV and CLAVEEVSL restricted to A*02:01, QEAIQDLCL to B*40:01, and LAVEEVSL to C*03:04, by multiple algorithms. These results support their potential relevance for further functional validation in the context of AML immunotherapy.

**Table 3:** *In silico* predicted binding affinities for neoepitopes derived from NPM1 mutations A/D, DNMT3A R882H, and R882C.

Mutation	HLA allele	NetMHCpan 4.1b	SYFPEITHI	RankPep	IEDB
NPM1mut A/D	A*02:01	AIQDLCLAV (0.924, WB) CLAVEEVSL (1.430, WB)	AIQDLCLAV (24) CLAVEEVSL (26)	AIQDLCLAV (85 [64]) CLAVEEVSL (70 [64]) DLCLAVEEV (68 [64])	AIQDLCLAV (1.8) CLAVEEVSL (5.5)
NPM1mut A/D	B*40:01	QEAIQDLCL (0.297, SB)	QEAIQDLCL (24)	n.a.	QEAIQDLCL (0.63)
NPM1mut A/D	C*03:04	LAVEEVSL* (0.494, SB)	n.a.	No binder	LAVEEVSL* (0.31)
NPM1mut A/D	B*18:01	QEAIQDLCL (1.466, WB)	No binder	n.a.	QEAIQDLCL (1.1)
DNMT3A R882H	B*40:01	TDVSNMSHL (1.895, WB)	No binder	n.a.	TDVSNMSHL (1.4)
DNMT3A R882C	B*40:01	TDVSNMSCL (1.895, WB)	No binder	n.a.	TDVSNMSCL (2.6)
DNMT3A R882H	C*03:04	MSHLARQRL (0.381, SB)	n.a.	No binder	MSHLARQRL (0.23)
DNMT3A R882C	C*03:04	MSCLARQRL (1.705, WB)	n.a.	No binder	YTDVSNMSCL** (0.86)

Predicted peptide–MHC-binding affinity is given in brackets behind the respective peptide. For RankPep, high binding affinities of peptides to MHC I are assumed when scores are higher than the binding threshold (values given in square brackets). For NetMHCpan4.1, SB denotes strong binder; WB, weak binder. n.a., not available. Point mutations are shown in bold red; *8-mer. **10-mer.

## 4 Discussion

Approximately 15 years have passed since the discovery of NPM1-mutated AML, and there is still no consensus on how to treat this type of AML. NPM1 represents the most frequently mutated gene in AML, and it is considered to be a good prognostic marker. NPM1-mutated AML has a favorable prognosis and is defined as a distinct leukemia entity in the World Health Organization classification. Mutated NPM1 is a promising and potential target for NPM1-mutated AML because the NPM1-mutated protein does not exist in normal tissues, and therefore, it is an ideal leukemia-specific antigen (LSA). It has been suggested that NPM1mut-derived LSAs can induce an anti-NPM1mut immune response by activating antigen-specific cytotoxic T cells. This leads to the hypothesis that individuals with suitable HLA types might be less prone to develop NPM1-mutated AML (23). Kuzelova et al. (2018) identified underrepresented HLA class I molecules, mainly A*02, B*07, B*40, and C*07:01 in a large cohort of 398 patients with NPM1-mutated AML compared with the normal frequencies obtained from www.allelefrequencies.net for healthy populations (23). In the present study, we analyzed the HLA distribution in 1,689 AML patients, with 477 AML patients positive for NPM1mutA/D, to validate these findings and, to our knowledge, for the first time, the HLA distribution of AML patients positive for DNMT3A R882H or R882C. Second, we aimed to confirm the potential relevance of NPM1 mutations as a specific anti-tumor target for TCR-based immunotherapy. Even without considering the mutation status of patients with AML at all, we observed significant differences in the frequencies of B*18:01 (p < 0.001), B*40:01 (p = 0.015), and C*03:04 (p = 0.02). For B*40:01 and C*03:04, the reduced frequencies can be revealed most probably due to the large proportion of patients with NPM1mut and/or DNMT3Amut within this “all AML patients” group in the present cohort (i.e., patients with NPM1mutA/D AML n=477 of 1,689) because for this NPM1mutA/D AML patient group, an even more pronounced statistically significant difference could be detected (see **Figure 3**). We did not find significant differences in the frequencies of B*07 and C*07:01. Not confirming data from Kuzelova et al. for the latter two HLA alleles could be explained at least partially by the following two differences: The reference cohort of the present study is a group of 51,890 healthy individuals HLA typed at DKMS (Germany), whereas Kuzelova et al. have used reference values obtained from www.allelefrequencies.net with HLA-frequency data of 11,407 individuals from Germany and 5,099 individuals from the Czech Republic. Furthermore, we used a two-field resolution in HLA typing, whereas the former study compared HLA allele frequencies typically only at one-field resolution.

However, we could confirm a significant decrease in A*02:01 frequency when focusing on AML patients with NPM1mutA/D (n = 477) compared to healthy individuals (39.0% vs. 48.1%, p < 0.001). HLA-A*02:01 is prevalent in 48.1% of healthy individuals in our cohort of 51,890 healthy individuals, and 49.9% of 11,407 German individuals obtained from www.allelefrequencies.net are A*02:01pos (**Table 4**). As approximately half of the Caucasian people carry A*02:01, it is a promising allelic group for the development of antigen-specific T cell-based immunotherapy. All four tested *in silico* peptide prediction online tools (NetMHCpan version 4.1b, SYFPEITHI, RankPep, and IEDB) forecasted two peptides with the best scores, namely, CLAVEEVSL and AIQDLCLAV, derived from type A/D mutated NPM1 protein, which efficiently bind to A*02:01, as previously published by several authors (9, 23, 37–39). To our knowledge, the validation of surface presentation on primary AML blasts of the peptide AIQDLCLAV is still lacking, whereas the peptide CLAVEEVSL has been shown to be presented on the surface of AML cell lines and even of A*02:01pos primary AML cells by mass spectrometry in independent studies (38, 40). Also, specific cytotoxic T-cell responses against these two NPM1 mutant peptides were detected in NPM1-mutated AML patients (9, 38, 41). Taken together (attractiveness, unique character, and suitability), these two peptides are very promising targets for AML immunotherapy. Besides A*02:01, we could confirm a reduced frequency of B*40:01 in AML patients with NPM1mut (4.0% vs. 10.2%, p < 0.001), in concordance with Kuzelova and colleagues (23, 42). In addition to the observed reduction of frequency of these two HLA alleles, we also observed a significantly lower frequency of B*15:01 (9.6% vs. 14.4%, p = 0.04) and C*03:04 (8.2% vs. 15.9%, p<0.001) in AML patients with NPM1mutA/D compared to healthy individuals (**Table 4**). A reduction of B*40:01 and C*03:04 can even be found when comparing the frequencies of AML patients with NPM1mutA/D versus AML patients w/o NPM1mutA/D or NPM1mut^rare^. This observation can be interpreted as a further hint for the existence of immunogenic NPM1-derived peptides. Actually, two strong binder peptides, QEAIQDLCL for HLA-B*40:01 and LAVEEVSL for HLA-C*03:04, were predicted *in silico* by at least two of the four mentioned peptide prediction software programs (**Table 3**), as formerly published by Kuzelova (42). To our knowledge, the respective peptides have not been identified by mass spectrometry, and no specific T cells have been found and isolated. It would be worthwhile to investigate peptide presentation of QEAIQDLCL and LAVEEVSL in B*40:01- or C03:04-positive patients with NPM1mut AML as well as to strive for the detection of respective T-cell responses.

**Table 4:** Overview of significant changes in HLA allele frequencies.

HLA allele	Freq in control	Freq in all AML pts	p-Value	Freq in pts with NPM1mutA/D	p-Value
A*02:01	48.1%	45.5%	0.42	39.0%	<0.001
B*15:01	14.3%	13.3%	1	9.6%	0.04
B*18:01	9.3%	12.3%	<0.001	12.6%	0.16
B*40:01	10.2%	7.8%	0.015	4.0%	<0.001
C*03:04	15.9%	13.1%	0.02	8.2%	<0.001

Freq, frequency; pts, patients; mut, mutation.

A reason for not fully confirming the results of Kuzelova could be that the reference cohort was a group of 51,890 healthy individuals HLA typed at DKMS (Germany) in this study, whereas in the former study, HLA-frequency data of 11,407 individuals from Germany and 5,099 individuals from the Czech Republic obtained from the www.allelefrequencies.net were used as reference values.

Results from the “What is the driver?” section point at a major contribution of B*40:01. Although the group is small (n = 21), there is nearly no reduction of the frequencies in patients who are positive for C*03:04 and negative for B*40:01. This suggests that the presence of C*03:04 with an absence of B*40:01 at the same time has no protective effect anymore. Another argument supporting the theory that it is HLA-B*40:01 that mediates the protective effect and not the genetically linked C*03:04 comes with the fact that HLA-C*03:03 and HLA-C*03:04 have highly similar peptide-binding motifs (43), while the protective effect has only been found for HLA-C*03:04, but not for HLA-C*03:03, suggesting that it is none of the two C alleles, but rather the linked B allele, that can efficiently present immunogenic peptides.

All peptide lengths (from 8- to 14-mer) were checked for the two HLA class I alleles using the last 21 C-terminal aa of mutated NPM1 protein and the surrounding 20 aa of position 882H of the mutated DNMT3A by NetMHCpan-4.1b, and for both HLA alleles, only one peptide was predicted to be a strong binder. However, using the whole mutated NPM1 sequence type A as input to find peptides with high immunogenicity, many more good binders for B*40:01 (14 strong binders and 34 weak binders, in total 48 binders) than for C*03:04 (5 strong binders and 16 weak binders, in total 21 binders) could be found. This is in agreement with the hypothesis that B*40:01 has the main influence on the protective effect for AML patients because of the intracellular delocalization of the mutated NPM1 in the cytoplasm. This is reasonable on the assumption that the exposition of mutated NPM1-derived peptides on the cell surface is increased in AML cells, whereas the wild-type form is primarily found in the nucleoli (23).

Kuzelova et al. (2018) recently identified a decreased HLA class I expression in AML samples with mutated NPM1 in the absence of a DNMT3A mutation (44). The effect of NPM1 mutation regarding HLA class I expression on the cell surface was prevented by a concurrent DNMT3A mutation. In other words, HLA class I expression in the double-mutated group is similarly high like in the unmutated group. Therefore, certain HLA alleles may only be protective in individuals with both two mutations (in NPM1 and DNMT3A), but not in NPM1 mutation co-occurring with DNMT3A wild type. As a consequence, we hypothesized that the observed frequency changes for B*40:01 and C*03:04 (and A*02:01, B*15:01) may be more pronounced in double mutants than in the “only NPM1mut” group because the HLA class I molecules remain on the surface, and an immune response by cytotoxic T cells can take place. Another finding, which might be supportive to the theory that NPM1- and DNMT3A-double mutated AML might be more prone to immunosurveillance, was published by Severens et al. (45) who demonstrated that NPM1-mutated AML can be divided into five clusters based on different gene expression profiles. Similar to Kuzelova et al., the authors demonstrated a low expression of HLA classes I and II in NPM1-mutated AML (with more primitive stem cell-like phenotypes) in which DNMT3A mutations are largely absent. However, in this cluster, a very distinct mutation profile is apparent when compared to the other four clusters. Therefore, the protective effect of HLA-B*40:01 and C*03:04 may also be explained by immune responses against neoantigens encoded by co-mutations that are enriched in NPM1-mutated DNMT3A-non-mutated AML. Given the low number of cases in our cohort, only exploratory analyses could be performed. For descriptive purposes, we observed lower frequencies of HLA-B*40:01 in AML patients with co-occurring mutations in NPM1 (A or D) and DNMT3A (R882H or C) compared to patients with NPM1mutA/D and unmutated DNMT3A (n = 2 of 84 vs. n = 2 of 61, 3.2% vs. 3.3%). HLA-B*40:01 was present in 13 of 291 (4.5%, 95% CI: 2.1%–6.8%) NPM1 (A or D) mutated patients having no information about DNMT3A mutations and in 2 of 41 (4.8%) patients with mutated NPM1 (A or D) and with rare DNMT3A mutations. However, these analyses have to be considered with caution, and it would be worthwhile to test this hypothesis in a larger patient cohort.

To further explore, whether HLA class I alleles with highly similar peptide-binding motif (PBM) are associated with a protective effect for AML, we analyzed the HLA supertypes or PBM clusters. But there are no significant effects for both HLA clusters. In the B44 supertype (PBM8-cluster), both HLA alleles, B*40:01 and B*18:01, are included. They both contribute to opposite effects. Interestingly, they are supposed to bind the same peptide (QEAIQDLCL, **Table 3**), and still, one appears to be protective (B*40:01) and the other as a risk factor (B*18:01), possibly due to the different HLA types conveying binding to different immune receptors such as TCRs, inhibitory or activating KIRs, and others. The opposite effect, as shown for B*40:01 and B*18:01, may provide an explanation why no effect can be detected for the B44 supertype or PBM8 cluster (both including B*40:01 and B*18:01) anymore. The C03 supertype group or the PBM18 cluster was compared with the C03 supertype(PBM18 cluster) without C*03:04. A tendency of reduction was observed in AML patients with NPM1mutA/D, but the reduction was not significant providing further evidence of a protective effect of HLA-C*03:04.

Additionally, a contribution to an anti-leukemic effect of the activating KIR2DS2 and KIR2DS3, both recognizing HLA-C-alleles of the C1-KIR allele group, remains to be shown as soon as NPM1-derived peptides are shown to be specifically mediating activating signals to NK cells, as described for KIR2DS4 and peptides presented via HLA-C*05:01 (46).

Various studies have shown by mass spectrometry that AVEEVSLRK is HLA presented on NPM1-mutated AML (38, 40, 47). AVEEVSLRK binds strongly to A*11:01 and is predicted to bind to A*03:01 as well (48). It has been reported to be an immunogenic 9-mer peptide derived from the C-terminus of the NPM1-mutated protein (41). Independent of the type of the insertion of 4 base pairs between nucleotides 960 and 961, mutations A, B, D, and some rare mutations (e.g., DD1–DD8) result in the elongation of the NPM1 protein with the same aa changes at the C-terminus. Therefore, we combined AML patients with NPM1mutA/D with AML patients with NPM1mutB and with all rare mutations (NPM1mut^AVEEV^), which result in the abovementioned aa sequence and compared these with healthy controls. We found a reduced frequency of A*11:01 in all AML patients with NPM1mut^AVEEV^, when compared to the control group, but the detected difference is not significant (n = 50 of 553 and n = 5,198 of 51,890, 9.0% vs. 10.0%, p = 0.49). The uncertainty that has to be taken into account (95% CI for difference: −3.4%–2.6%) points to the possibility that the reduction in HLA frequencies could also be more pronounced. Otherwise, this result could also suggest that A*11:01 might not be protective regarding the development of NPM1mut^AVEEV^ AML, although peptide presentation and T-cell recognition have been shown in other studies. So far, the cytotoxic effect of AVEEVSLRK-specific T cells could be demonstrated for patient AML cells *in vitro*, but T cells failed to induce an *in vivo* anti-tumor response in an aggressive mouse model for human AML (41, 48). Furthermore, it could be speculated that an ongoing T-cell response against A*11:01-presented AVEEVSLRK might not be efficient to eliminate developing AML cells due to (i) escape mechanisms of the AML cells, which might be efficient, especially for A*11:01 or A*11:01-presented peptides, (ii) gene variants or genetic polymorphisms, which are in a linkage disequilibrium with HLA-A*11:01, e.g., HLA haplotypes, including HLA-class 2 genes, cytokines, or other factors encoded in the MHC locus, which are, more or less, efficient in priming or maintaining an immune response against NPM1mut, or (iii) other unknown reasons.

## 5 Conclusion

In summary, (i) we were able to confirm the results of the study by Kuzelova et al. (2018) showing that the frequencies of A*02:01, B*40:01, and C*03:04 are lower in AML patients with NPM1 mutations A/D in an independent cohort, including 1,689 AML patients and a healthy control group with more than 50,000 individuals. As a consequence, (ii) our analysis strongly indicates that an efficient immune response against mutated NPM1 can be induced in patients bearing suitable alleles. Furthermore, (iii) using four different *in silico* epitope prediction software tools, we were able to define candidate peptides derived from mutated NPM1 that might be recognized by specific cytotoxic T cells for the above protective HLA class I alleles. Taken together, (iv) these findings suggest further efforts to identify and isolate HLA-restricted NPM1mut-specific cytotoxic T cells to sequence the specific T-cell receptor. This sequence information can eventually be exploited to genetically modify T cells for immunotherapy of AML patients.

## Data Availability

The datasets presented in this article are not readily available because they contain information that could compromise patient privacy. Requests to access the datasets should be directed to the corresponding author, Elke Rücker-Braun (elke.ruecker-braun@ukdd.de). Requests will be checked carefully and data will be made available by the authors without undue reservation.

## References

[1] Barrett AJ. Acute myeloid leukaemia and the immune system: implications for immunotherapy. Br J Haematol. (2020) 188:147–58. doi: 10.1111/bjh.v188.1 31782805

[2] Falini B Lenze D Hasserjian R Coupland S Jaehne D Soupir C. Cytoplasmic mutated nucleophosmin (NPM) defines the molecular status of a significant fraction of myeloid sarcomas. Leukemia. (2007) 21:1566–70. doi: 10.1038/sj.leu.2404699 17443224

[3] Falini B Nicoletti I Martelli MF Mecucci C. Acute myeloid leukemia carrying cytoplasmic/mutated nucleophosmin (NPMc+ AML): biologic and clinical features. Blood. (2007) 109:874–85. doi: 10.1182/blood-2006-07-012252 17008539

[4] Falini B Martelli MP Bolli N Sportoletti P Liso A Tiacci E. Acute myeloid leukemia with mutated nucleophosmin (NPM1): is it a distinct entity? Blood. (2011) 117:1109–20. doi: 10.1182/blood-2010-08-299990 21030560

[5] Bacher U Porret N Joncourt R Sanz J Aliu N Wiedemann G. Pitfalls in the molecular follow up of NPM1 mutant acute myeloid leukemia. Haematologica. (2018) 103:e486–e8. doi: 10.3324/haematol.2018.192104 PMC6165800 29903758

[6] Merlo MEB Mallardo M Luzi L De Conti G Caprioli C Hillje R. Enforcement of stem-cell dormancy by nucleophosmin mutation is a critical determinant of unrestricted self-renewal during myeloid leukemogenesis. Haematologica. (2025). doi: 10.3324/haematol.2024.286577 PMC12399960 40079083

[7] Falini B Mecucci C Tiacci E Alcalay M Rosati R Pasqualucci L. Cytoplasmic nucleophosmin in acute myelogenous leukemia with a normal karyotype. N Engl J Med. (2005) 352:254–66. doi: 10.1056/NEJMoa041974 15659725

[8] Becker H Marcucci G Maharry K Radmacher MD Mrozek K Margeson D. Favorable prognostic impact of NPM1 mutations in older patients with cytogenetically normal *de novo* acute myeloid leukemia and associated gene- and microRNA-expression signatures: a Cancer and Leukemia Group B study. J Clin Oncol. (2010) 28:596–604. doi: 10.1200/JCO.2009.25.1496 20026798 PMC2815994

[9] Greiner J Ono Y Hofmann S Schmitt A Mehring E Gotz M. Mutated regions of nucleophosmin 1 elicit both CD4(+) and CD8(+) T-cell responses in patients with acute myeloid leukemia. Blood. (2012) 120:1282–9. doi: 10.1182/blood-2011-11-394395 22592607

[10] Chang JH Olson MO. Structure of the gene for rat nucleolar protein B23. J Biol Chem. (1990) 265:18227–33. doi: 10.1016/S0021-9258(17)44742-9 2211699

[11] Cordell JL Pulford KA Bigerna B Roncador G Banham A Colombo E. Detection of normal and chimeric nucleophosmin in human cells. Blood. (1999) 93:632–42. doi: 10.1182/blood.V93.2.632 9885226

[12] Borer RA Lehner CF Eppenberger HM Nigg EA. Major nucleolar proteins shuttle between nucleus and cytoplasm. Cell. (1989) 56:379–90. doi: 10.1016/0092-8674(89)90241-9 2914325

[13] Federici L Falini B. Nucleophosmin mutations in acute myeloid leukemia: a tale of protein unfolding and mislocalization. Protein Sci. (2013) 22:545–56. doi: 10.1002/pro.v22.5 PMC3649256 23436734

[14] Chen W Rassidakis GZ Medeiros LJ. Nucleophosmin gene mutations in acute myeloid leukemia. Arch Pathol Lab Med. (2006) 130:1687–92. doi: 10.5858/2006-130-1687-NGMIAM 17076533

[15] Su M Cheng H Cheng T. Preleukemic stem cells: leave it or not? Blood Sci. (2020) 2:54–8. doi: 10.1097/BS9.0000000000000042 PMC8975042 35402817

[16] Bullinger L Dohner K Dohner H. Genomics of acute myeloid leukemia diagnosis and pathways. J Clin Oncol. (2017) 35:934–46. doi: 10.1200/JCO.2016.71.2208 28297624

[17] Gaidzik VI Schlenk RF Paschka P Stolzle A Spath D Kuendgen A. Clinical impact of DNMT3A mutations in younger adult patients with acute myeloid leukemia: results of the AML Study Group (AMLSG). Blood. (2013) 121:4769–77. doi: 10.1182/blood-2012-10-461624 23632886

[18] Xu F Mao C Ding Y Rui C Wu L Shi A. Molecular and enzymatic profiles of mammalian DNA methyltransferases: structures and targets for drugs. Curr Med Chem. (2010) 17:4052–71. doi: 10.2174/092986710793205372 PMC3003592 20939822

[19] Welch JS Ley TJ Link DC Miller CA Larson DE Koboldt DC. The origin and evolution of mutations in acute myeloid leukemia. Cell. (2012) 150:264–78. doi: 10.1016/j.cell.2012.06.023 PMC3407563 22817890

[20] Kohnke T Karigane D Hilgart E Fan AC Kayamori K Miyauchi M. DNMT3A(R882H) is not required for disease maintenance in primary human AML, but is associated with increased leukemia stem cell frequency. bioRxiv. (2024). doi: 10.1101/2024.10.26.620318 PMC12717831 41263425

[21] Eugster A Lindner A Heninger AK Wilhelm C Dietz S Catani M. Measuring T cell receptor and T cell gene expression diversity in antigen-responsive human CD4+ T cells. J Immunol Methods. (2013) 400-401:13–22. doi: 10.1016/j.jim.2013.11.003 24239865

[22] Wooldridge L Ekeruche-Makinde J van den Berg HA Skowera A Miles JJ Tan MP. A single autoimmune T cell receptor recognizes more than a million different peptides. J Biol Chem. (2012) 287:1168–77. doi: 10.1074/jbc.M111.289488 PMC3256900 22102287

[23] Kuzelova K Brodska B Schetelig J Rollig C Racil Z Walz JS. Association of HLA class I type with prevalence and outcome of patients with acute myeloid leukemia and mutated nucleophosmin. PloS One. (2018) 13:e0204290. doi: 10.1371/journal.pone.0204290 30557403 PMC6296532

[24] Spielau C Bunzel C Abert S Baldauf H Schmidt AH Schetelig J. The Collaborative Biobank (CoBi): Donor and recipient samples & data to facilitate future research on hematopoietic cell transplantation. Best Pract Res Clin Haematol. (2024) 37:101551. doi: 10.1016/j.beha.2024.101551 39098795

[25] Thiede C Koch S Creutzig E Steudel C Illmer T Schaich M. Prevalence and prognostic impact of NPM1 mutations in 1485 adult patients with acute myeloid leukemia (AML). Blood. (2006) 107:4011–20. doi: 10.1182/blood-2005-08-3167 16455956

[26] Stasik S Schuster C Ortlepp C Platzbecker U Bornhauser M Schetelig J. An optimized targeted Next-Generation Sequencing approach for sensitive detection of single nucleotide variants. Biomol Detect Quantif. (2018) 15:6–12. doi: 10.1016/j.bdq.2017.12.001 29349042 PMC5766748

[27] Lange V Bohme I Hofmann J Lang K Sauter J Schone B. Cost-efficient high-throughput HLA typing by MiSeq amplicon sequencing. BMC Genomics. (2014) 15:63. doi: 10.1186/1471-2164-15-63 24460756 PMC3909933

[28] Kim Y Ponomarenko J Zhu Z Tamang D Wang P Greenbaum J. Immune epitope database analysis resource. Nucleic Acids Res. (2012) 40:W525–30. doi: 10.1093/nar/gks438 PMC3394288 22610854

[29] Andreatta M Nielsen M. Gapped sequence alignment using artificial neural networks: application to the MHC class I system. Bioinformatics. (2016) 32:511–7. doi: 10.1093/bioinformatics/btv639 PMC6402319 26515819

[30] Peters B Sette A. Generating quantitative models describing the sequence specificity of biological processes with the stabilized matrix method. BMC Bioinf. (2005) 6:132. doi: 10.1186/1471-2105-6-132 PMC1173087 15927070

[31] Sidney J Assarsson E Moore C Ngo S Pinilla C Sette A. Quantitative peptide binding motifs for 19 human and mouse MHC class I molecules derived using positional scanning combinatorial peptide libraries. Immunome Res. (2008) 4:2. doi: 10.1186/1745-7580-4-2 18221540 PMC2248166

[32] Ip PP Nijman HW Daemen T. Epitope prediction assays combined with validation assays strongly narrows down putative cytotoxic T lymphocyte epitopes. Vaccines (Basel). (2015) 3:203–20. doi: 10.3390/vaccines3020203 PMC4494349 26343185

[33] Schmidt AH Baier D Solloch UV Stahr A Cereb N Wassmuth R. Estimation of high-resolution HLA-A, -B, -C, -DRB1 allele and haplotype frequencies based on 8862 German stem cell donors and implications for strategic donor registry planning. Hum Immunol. (2009) 70:895–902. doi: 10.1016/j.humimm.2009.08.006 19683023

[34] Crivello P Arrieta-Bolanos E He M Wang T Fingerson S Gadalla SM. Impact of the HLA immunopeptidome on survival of leukemia patients after unrelated donor transplantation. J Clin Oncol. (2023) 41:2416–27. doi: 10.1200/JCO.22.01229 PMC10150892 36669145

[35] Sidney J Peters B Frahm N Brander C Sette A. HLA class I supertypes: a revised and updated classification. BMC Immunol. (2008) 9:1. doi: 10.1186/1471-2172-9-1 18211710 PMC2245908

[36] Gfeller D Bassani-Sternberg M. Predicting antigen presentation-what could we learn from a million peptides? Front Immunol. (2018) 9:1716. doi: 10.3389/fimmu.2018.01716 30090105 PMC6068240

[37] Liso A Colau D Benmaamar R De Groot A Martin W Benedetti R. Nucleophosmin leukaemic mutants contain C-terminus peptides that bind HLA class I molecules. Leukemia. (2008) 22:424–6. doi: 10.1038/sj.leu.2404887 17690700

[38] van der Lee DI Reijmers RM Honders MW Hagedoorn RS de Jong RC Kester MG. Mutated nucleophosmin 1 as immunotherapy target in acute myeloid leukemia. J Clin Invest. (2019) 129:774–85. 10.1172/JCI97482 PMC6355238 30640174

[39] Rucker-Braun E Link CS Schmiedgen M Tunger A Vizjak P Teipel R. Longitudinal analyses of leukemia-associated antigen-specific CD8(+) T cells in patients after allogeneic stem cell transplantation. Exp Hematol. (2016) 44:1024–33 e1. 27473564 10.1016/j.exphem.2016.07.008

[40] Narayan R Olsson N Wagar LE Medeiros BC Meyer E Czerwinski D. Acute myeloid leukemia immunopeptidome reveals HLA presentation of mutated nucleophosmin. PloS One. (2019) 14:e0219547. doi: 10.1371/journal.pone.0219547 31291378 PMC6619824

[41] Forghieri F Riva G Lagreca I Barozzi P Vallerini D Morselli M. Characterization and dynamics of specific T cells against nucleophosmin-1 (NPM1)-mutated peptides in patients with NPM1-mutated acute myeloid leukemia. Oncotarget. (2019) 10:869–82. doi: 10.18632/oncotarget.26617 PMC6368236 30783516

[42] Kuzelova K Brodska B Fuchs O Dobrovolna M Soukup P Cetkovsky P. Altered HLA class I profile associated with type A/D nucleophosmin mutation points to possible anti-nucleophosmin immune response in acute myeloid leukemia. PloS One. (2015) 10:e0127637. doi: 10.1371/journal.pone.0127637 25992555 PMC4439052

[43] Rasmussen M Harndahl M Stryhn A Boucherma R Nielsen LL Lemonnier FA. Uncovering the peptide-binding specificities of HLA-C: a general strategy to determine the specificity of any MHC class I molecule. J Immunol. (2014) 193:4790–802. doi: 10.4049/jimmunol.1401689 PMC4226424 25311805

[44] Kuzelova K Brodska B Markova J Petrackova M Schetelig J Ransdorfova S. NPM1 and DNMT3A mutations are associated with distinct blast immunophenotype in acute myeloid leukemia. Oncoimmunology. (2022) 11:2073050. doi: 10.1080/2162402X.2022.2073050 35558161 PMC9090295

[45] Severens JF Karakaslar EO van der Reijden BA Sanchez-Lopez E van den Berg RR Halkes CJM. Mapping AML heterogeneity - multi-cohort transcriptomic analysis identifies novel clusters and divergent ex-vivo drug responses. Leukemia. (2024) 38:751–61. doi: 10.1038/s41375-024-02137-6 38360865

[46] Sim MJW Rajagopalan S Altmann DM Boyton RJ Sun PD Long EO. Human NK cell receptor KIR2DS4 detects a conserved bacterial epitope presented by HLA-C. Proc Natl Acad Sci U S A. (2019) 116:12964–73. doi: 10.1073/pnas.1903781116 PMC6601252 31138701

[47] Nelde A Schuster H Heitmann JS Bauer J Maringer Y Zwick M. Immune surveillance of acute myeloid leukemia is mediated by HLA-presented antigens on leukemia progenitor cells. Blood Cancer Discov. (2023) 4:468–89. doi: 10.1158/2643-3230.BCD-23-0020 PMC10618727 37847741

[48] van der Lee DI Koutsoumpli G Reijmers RM Honders MW de Jong RCM Remst DFG. An HLA-A*11:01-binding neoantigen from mutated NPM1 as target for TCR gene therapy in AML. Cancers (Basel). (2021) 13:1–17. doi: 10.3390/cancers13215390 PMC8582585 34771556

