# Supplementary material for: Protective effects for HLA-B*40:01 and C*03:04 in NPM1-mutated AML: result of a large HLA association study
Source: Front Immunol. 2025 Jun 10;16:1571508. doi: 10.3389/fimmu.2025.1571508 (PMC12185502; doi:10.3389/fimmu.2025.1571508)

Supplementary Material

1 Supplementary Figures

1.1 Figure S1. Frequencies of the ten most frequent HLA-A, -B and -C alleles in AML patients with DNMT3A R882H mutation (grey bars) and without DNMT3A R882H (orange bars) and in healthy donors (blue bars).

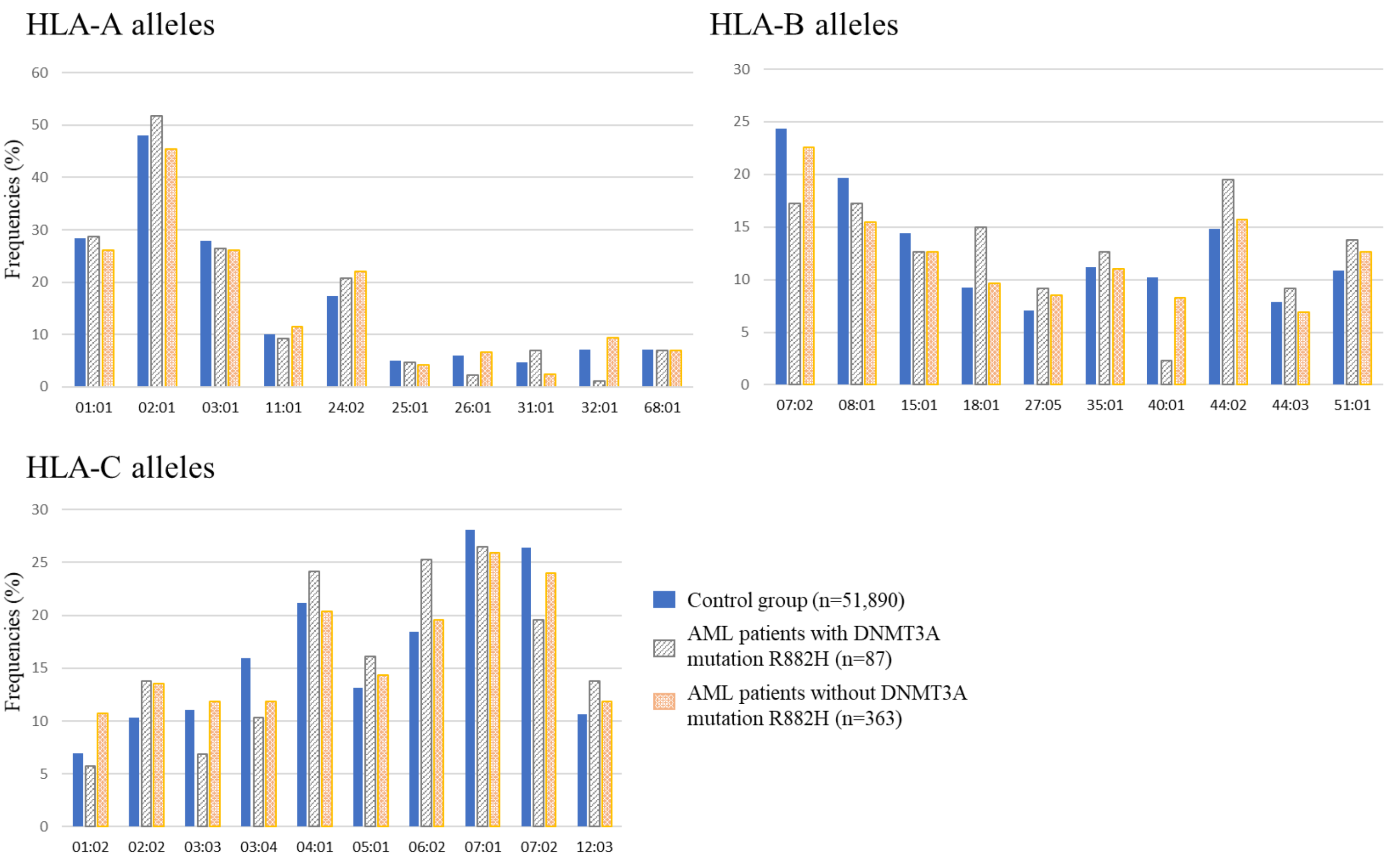

1.2 Figure S2. Frequencies of the ten most frequent HLA-A, -B and -C alleles in AML patients with both DNMT3A mutations, R882H and R882C, (grey bars) and healthy donors (blue bars).

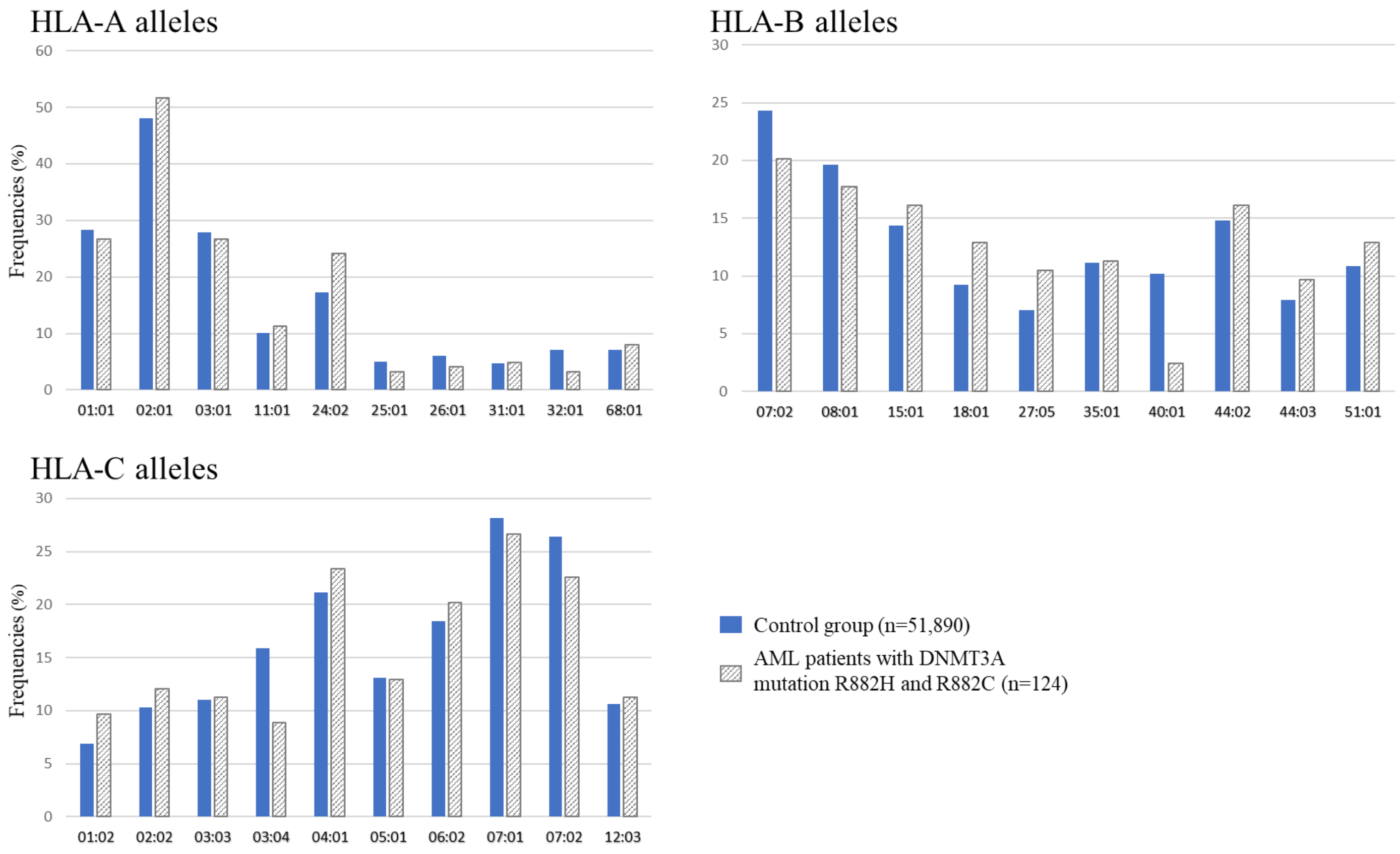

Supplement: Supplementary file 1 [file DataSheet1.pdf]
